# Progress in murine models of ruptured abdominal aortic aneurysm

**DOI:** 10.3389/fcvm.2022.950018

**Published:** 2022-08-12

**Authors:** Li Yin, Eric William Kent, Bowen Wang

**Affiliations:** Department of Surgery, School of Medicine, University of Virginia, Charlottesville, VA, United States

**Keywords:** mouse model, abdominal aortic aneurysm, rupture aneurysm, elastase, AngII

## Abstract

Abdominal aortic aneurysm (AAA) is a focal dilation of the aorta that is prevalent in aged populations. The progressive and unpredictable expansion of AAA could result in aneurysmal rupture, which is associated with ~80% mortality. Due to the expanded screening efforts and progress in diagnostic tools, an ever-increasing amount of asymptomatic AAA patients are being identified yet without a cure to stop the rampant aortic expansion. A key barrier that hinders the development of effective AAA treatment is our incomplete understanding of the cellular and molecular basis of its pathogenesis and progression into rupture. Animal models provide invaluable mechanistic insights into AAA pathophysiology. However, there is no single experimental model that completely recapitulate the complex biology behind AAA, and different AAA-inducing methodologies are associated with distinct disease course and rupture rate. In this review article, we summarize the established murine models of ruptured AAA and discuss their respective strengths and utilities.

## Introduction

An abdominal aortic aneurysm, also known as AAA, is defined as a localized dilation of the abdominal aorta. The expansion involves all layers of the aortic wall. The risk factors for AAA include advanced age, male, smoking, hypertension, dyslipidemia, family history, other vascular aneurysms, other vascular diseases, atherosclerosis, and hypercholesterolemia (1–3). Current guidelines recommend a surveillance strategy for patients of small, asymptomatic AAAs with interval imaging. Surgical repair, including open surgery and endovascular aneurysm repair (EVAR), remains the only treatment for AAA (4).

The main complication of AAA is aortic rupture, which is responsible for about 200,000 deaths per year worldwide (5). The mortality associated with ruptured abdominal aortic aneurysm (rAAA) is still alarmingly high. Despite the progress in emergent surgical repairs, the in-hospital all mortality for rAAA patients remains over 50% in the USA (6). In the population-based Malmö Diet and Cancer Study (MDCS), the acute mortality, defined as death before reaching a hospital or during the first admission, was 34% for rAAA (7). Inasmuch as the high lethality of rAAA, significant efforts have been devoted to the development of robust prediction tools as well as pharmacotherapies for small, asymptomatic AAAs to prevent the deadly their deadly progressions toward rupture, yet none have demonstrated definitive clinical benefits. These pressing unmet needs testifies to the necessity for further elucidation of the cellular and molecular basis behind AAA pathogenesis as well as its uncontrolled progression into rupture.

Animal models provide invaluable insights into AAA pathophysiologies and help inform clinical studies concerning innovative therapeutics and diagnostics. Experimental models of AAA have been described in a wide range of species, including mice, rats, rabbits, swine, sheep, etc. Due to the multifactorial etiology of AAA, the aneurysm-inducing procedures vary greatly amongst different models. The majority of the existing models focus on the onset phase of AAA, featuring varying incidence rates, anatomical locations, lesion sizes, and disease courses. So far, the three most widely adopted AAA-inducing methods are angiotensin II (AngII) infusion, porcine pancreatic elastase perfusion, and calcium salt topical application. In recent years, β-aminopropionitrile (BAPN), an irreversible inhibitor of lysyl oxidase, becomes increasingly recognized as either a highly effective inducer of aortic aneurysm, dissection, and rupture in murine models—either standalone or in combination with other classic AAA-inducing procedures. These different animal models have been shown to resemble certain aspects of the cellular compositions and pathological changes of clinical AAA, as evidenced by recent transgenic and single-cell sequencing analysis. However, very few of the existing models could faithfully recapitulate the clinical features of rAAA. In fact, the majority of the aforementioned models either do not depict rupture events at all, or are predominantly associated with ruptures in the ascending or thoracic aortas. Additionally, the timing of rupture events differs significantly amongst these models, and the rupture rates have been inconsistent throughout different reports and models. The ideal rAAA model should provide consistent aneurysm formation in the appropriate anatomical location (i.e., infrarenal aortic segment) and consistent AAA rupture over a chronic course. With the emerging interest and research efforts dedicated to the propagation phase of AAA, it is of significant mechanistic and translational values to identify the appropriate experimental models that recapitulate the progressive AAA progression into rupture (8). Herein, we will review the murine models of rAAA and discuss their respective strengths and shortcomings for preclinical studies.

## Murine models with systemic agent administration

From a utility perspective, systematical administration of chemical agents is arguably the easiest fashion to establish AAA lesions. Typically, these agents are judiciously chosen based on their association with AAA's clinical risk factors. The most widely utilized ones are hypertension (e.g., AngII and high salt intake) and hypercholesterolemia (e.g., high-fat diet and ApoE/LDLr/PCSK9 loss-of-functions), which we will elaborate later. However, in comparison to the more technically challenging microsurgery-induced models, mice systemically administered with such AAA-inducing agents tend to show highly variable outcomes in terms of AAA incidence, size, location, and rupture rate. It is worth noting that both AngII- and high salt-based models feature both AAA as well as aortic aneurysm and dissection (AAD) in the ascending and thoracic aortas. Herein, we will solely focus on their relevance in rAAA.

### Angiotensin II (AngII)-induced rAAA in hypercholesterolemic mice

AngII infusion is one of the most popular methodology to induce AAA in rodents. This model was first reported 20 years ago by Daugherty et al. and has remained the mainstream for aneurysmal studies, particularly concerning ascending and thoracic aortic aneurysm and dissection. In this model, an Azlet osmotic pump will be implanted subcutaneously in mice that are susceptible to hypercholesterolemia (e.g., ApoE-/-), providing subcutaneous infusion of angiotensin II at a dose of 500 or 1,000 ng/kg/min for varying durations, ranging from 14 to 28 days. High-fat or regular diets will be provided during the induction period (9, 10). As aforementioned, this model is primarily focused on aortic aneurysm and dissection in the ascending and thoracic aortas, while the incidence of AAA has been highly variable and inconsistent across different studies (11). In lieu of other modifications, a hypercholesterolemic background is indispensable for the AngII infusion model. Aside from ApoE-/- mice, LDLr mutants have also been widely utilized for this model, albeit with smaller aneurysmal lesions (12). Lu et al. recently established an alternative approach to establish the hypercholesterolemic co-morbidity, in which a PCSK9 gain-of-function adeno-associated virus (AAV) was systemically administered. This new methodology has gained significant popularity as it bypasses the necessity for the ApoE or LDLr mutant alleles, which can be considerably cumbersome and time-consuming for studies that involve additional transgene alleles.

AngII+hypercholesterolemia-induced AAAs recapitulate certain key characteristics of human AAA, such as aortic vascular inflammation, macrophage recruitment, aneurysmal tissue remodeling, and most importantly, the possibility of aneurysmal rupture. Nevertheless, critical discrepancies have been noted in AngII+hypercholesterolemia-induced AAAs vs. typical clinical lesions (13). First of all, AngII-induced aneurysms in mice have been consistently observed to be located in the suprarenal aorta, while most human AAAs are located to infrarenal aorta (11, 14). These differences may be the result of the potential hemodynamic and anatomy differences, but the exact mechanism remains unclear. Several other chemical-induced AAA models also share such anatomical features with AngII models (11). The pathological features in AngII-induced aneurysms tissue varies spatially and temporally during AngII infusion (15). In the first few days of AngII infusion, the aorta lumen expanded rapidly and an overt thrombus exists outside the external elastic lamina in the adventitia (16, 17). The lumen diameter increases from 0.9 to 1.5 mm within a week (18, 19), resulting in a high rate of death attributed to aorta rupture in the first week and the death occurs less frequently in the following (20, 21). Finally, about 15% to 30% of mice develop abdominal or thoracic aortic rupture in 4 weeks, albeit the incidence rates vary significantly amongst reports. Some studies featuring prolonged AngII infusion observed a gradual increase in aorta lumen dimension for ~3mm after 3 months in LDLr-/- or ApoE-/- mice, which may not lead to a significant increase in rupture rate. The most important difference is that AngII models induce more aortic dissection than AAA (16). AngII-induced aneurysms walls present intramural hematomas and intima tears, both typical characteristics of aortic dissection; in contrast, human AAA lesions feature significant less dissection but more intraluminal thrombosis (11, 16, 22, 23). Therefore, it has been widely argued that AngII-infused mice are more clinically relevant for studying aortic dissections than AAA (22).

### rAAA models induced by AngII+ β-aminopropionitrile (BAPN)

BAPN is an inhibitor of lysyl oxidase and has been reported to disrupt the aortic integrity of collagen and elastin structures, mimicking the aging-associated aortic stiffness. The first documentation of BAPN in AAA was by Kanematsu et al., who reported the robust induction of rAAA in wildtype c57/b6 mice without hypercholesterolemia. BAPN (150 mg/kg per day) infusion was administered *via* osmotic pumps implanted subcutaneously for the first 2 weeks with 1000ng/kg/min AngII infusion throughout 6 weeks. The mice in this study developed thoracic and abdominal aortic aneurysms (38–50% and 30–49 %, respectively) (24). About 33% (16 of 45) died from ruptured aortic aneurysms, including thoracic aneurysms, abdominal aneurysms, and dissecting aneurysms. Aside from BAPN infusion *via* the Azlet osmotic pumps (25, 26), others have reported BAPN systemic administration *via* drinking water modification or gastric lavage (27–31), at a concentration of 0.1~0.6%. is more popular since it's convenient (28, 30, 32–34).

AngII+BAPN-induced aneurysms have a similar histological characteristics to AngII-induced aneurysms, including ECM degeneration, SMC apoptosis, and aortic dissections. Although AngII+BAPN model presents some aortic dissection aneurysms and aortic dissection, this model leads to a higher incidence of AAA than the conventional AngII models, thereby making it a more appropriate model for studying rAAA (28, 30, 32–35). As characterized by Fashandi et al., the combination of AngII and low dose BAPN constitutes a reproducible model of aortic aneurysm rupture(32). In this study, ApoE -/- mice were given AngII at 1,500–2,000ng/kg/min and 0.2% BAPN dissolved in drinking water, resulting in 93%/79%/79% mice developing AAAs/thoracic aneurysms/ruptures over the course of 4 weeks, respectively. Additional studies further demonstrated the robustness of the BAPN-supplemented models in inducing rAAAs in lieu of hypercholesterolemic backgrounds, which provides significant benefits for transgenic studies (36). It is worth noting that BAPN alone has also been utilized to induce aneurysm rupture; however, similar to the classic AngII models, high dose BAPN administration primarily affected the ascending and thoracic aortic segments instead of abdominal aortas (27, 37).

### rAAA exacerbated by high salt

Despite inconsistent reports, epidemiological studies suggests a potential association between high salt consumption and AAA risk (38, 39). In murine models, however, high salt intake alone failed to induce aneurysm formation. By combining high salt with other experimental conditions, rAAA can be robustly induced with high incidence rate within 14 days. Liu et al. implanted deoxycorticosterone acetate pellets with a high salt diet and aldosterone infusion with a high salt diet in male mice at a relatively older age (e.g., 10–12 months old), inducing aneurysm lesions similar to the conventional AngII model (40). In a separate study, high-salt challenge significantly increased the rupture risks of both thoracic and abdominal aortic aneurysm in an AngII+BAPN model. Additional studies are needed to further characterize the impact of high salt in rAAA models.

## Rodent models induced by localized challenges

### Elastase-induced AAA models

Elastase-induced model, including intraluminal perfusion and topical application, is the second most commonly used model in AAA studies in both mice and rats (41–44). In the classic intraluminal model, the abdominal aortas are typically filled with 0.414 U/mL Type I porcine pancreatic elastase using a syringe pump calibrated to 100 mmHg (42). After 5 min perfusion, the perfused aortas typically dilate by ~50–70%. In this model, porcine pancreatic elastase is pressure-perfused into the aorta, which causes an immediate increase in diameter due to the inflation pressure and typically results in an aneurysm by the second week, the aorta diameter may reach 300–400% of the initial size. This procedure also works on wild-type mice (45).

One key disadvantage of the intraluminal method lies in its technical difficulty. Indeed, the microsurgical procedure involves exposure of the infrarenal aorta, temporary occlusion, arteriotomy creation, catheterization, and aorta repairing. Creation of this model requires advanced surgical skills, and there is a substantial learning curve. Because of the technical challenge in mice, elastase perfusion procedures are alternatively performed in rats; however, considering the relative paucity of transgenic rats for genes of interest, mice are still the indispensable species of choice. Alternatively, peri-aortic topical elastase application has been recently established as an alternative approach to robustly induce aneurysms resulting in AAA (43, 46, 47). As described by Bhamidipati et al., ~10 μL 100% porcine pancreatic elastase was topically applied to abdominal aortic segments for 10 min. After 2 weeks, the aortic diameter increased by 80% with 60% incidence (47). A later study by Lu et al. reported better outcomes with technical optimizations. For example, they applied 5 μL of the active form of elastase (10.3 mg protein/mL, 5.9 U/mg protein) topically to the adventitia of the infrarenal aorta for 5 min (an anatomical recess structure described as “boat” was utilized to “bath” the aforementioned abdominal aortic segments) (43). Aneurysms developed in 87.5% of the mice after 2 weeks; and fueled by BAPN water modification (elaborated later), the aneurysmal diameter continued to progress over the course of 100 days, recapitulating key clinical sequelae of AAA such as rupture and intraluminal thrombosis. Further studies demonstrate comparable macro and microscopic disease features between the intraluminal and topic models using elastase, thereby significantly reducing the bar for wide adoption by researchers (46, 48, 49).

### rAAA models enabled by the combination of elastase and systemic chemical administration

Compared to the dissection in AngII-based models, elastase-induced models feature aortic enlargements in true lumens, more marked inflammation, and accurate anatomical lesion location (i.e., infrarenal), all of which are important features in clinical AAA. A prominent problem with the conventional elastase model lies in its lack of rupture. Additionally, while elastase-induced aneurysms can reach a rather large size, they tend to reach plateau after the first 2 weeks. However, it is worth noting that the recent introduction of BAPN significantly alleviates these forgoing concerns. Several models have been reported by combining elastase topical application with BAPN to create a more desirable rupture model. In a recent study, the topical elastase+BAPN-induced AAA lesions was characterized with high-resolution ultrasound imaging, clearly demonstrating the continuous aneurysmal expansion fueled by BAPN(50). Similarly, Lu et al. combined oral BAPN administration and topical elastase application to recapitulate the chronic progression of AAA toward rupture beyond the commonly used 4-week window (43). The mice were given 0.2% BAPN water and adventitia elastase, and the AAA formation rate reached 95% by day 14. In addition to profound AAA lesions, other sequelae, such as thrombosis, irregular shape, thin areas on aneurysm and/or iliac, and renal arteries involvement, were observed, all of which are characteristics of advanced-stage AAA. Moreover, 46.2% of the mice died of AAA rupture before the end of the experiment, including 15.4% (2/13) acute rupture and 30.8% during the chronic, advanced stage (beyond 60 days post elastase challenge). In a separate study, Yue et al. combined intraluminal elastase perfusion (2.0 U/mL elastase at 100 mmHg for 15 mmin) with AngII infusion (1,000 ng/kg/min through osmotic pumps) (51). Similar to BAPN, AngII infusion increased the rupture rate of elastase-induced AAA lesions, reaching around 60% by 4 weeks post procedure.

### Calcium chloride induced model

Adventitia application of calcium salts, usually calcium chloride or calcium phosphate, is another accepted way to induce AAA (52, 53). The diameter of calcium salts induced aneurysms is typically smaller than other models (54, 55). Calcium salts induced aneurysms present inflammation, angiogenesis, elastin degradation, and calcification as found in human AAA, but they do not rupture (55, 56).

## Other models of rAAA

An increasing number of modified models have been reported based on the classical AAA models, *via* introducing new chemical agents or microsurgical manipulations (57–59). Lareyre et al. conducted a topical application elastase model, and the mice were injected mouse anti-mouse TGF-β three times a week, promoting severe AAA (59). Other notable modification of classic models include retroperitoneal surgical approaches, outflow modulations, and extended elastase perfusion to the juxtarenal and aortoiliac segments (57, 58). Additionally, a hypoperfusion model of the adventitial vasa vasorum in rats was recently described, but this procedure has not been replicated in murine models and the rupture risk is suboptimal and inconsistent (60–62).

## Closing remarks

**Figure 1:**
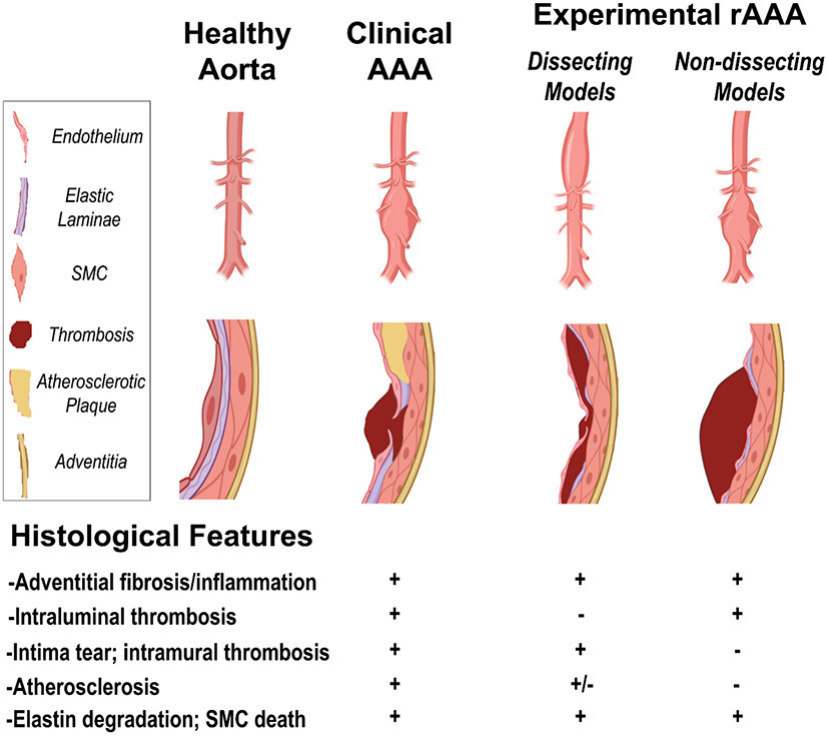
The morphological and histological features in clinical vs. experimental models of rAAA (created with BioRender.com).

An increasing number of murine models are being developed and characterized to facilitate the mechanistic and therapeutic endeavors concerning rAAA. However, there are several key barriers intrinsic to the existing models, including the inconsistent aneurysm incidence and suboptimal penetrance (particularly for systemically induced models), minimal rupture risks in abdominal aortas (e.g., unmodified elastase model and conventional AngII model), technical difficulties (e.g., intraluminal elastase perfusion model), etc. Indeed, no single experimental model described above fully recapitulate the pathophysiologies of AAA in patients. Figure 1 summarizes the morphological and histological features that are either shared or distinctive in clinical vs. experimental models. Combining different AAA-inducing approaches may offer an avenue to better recapitulate the complex pathophysiolgies of AAA. For example, the combination of intraluminal elastase perfusion and systemic AngII infusion was recently shown to effectively induce rAAA within 4 weeks; and in stark contrast to the conventional AngII-induced AAA models, the aneurysmal lesions in this modified model were primarily located in the infrarenal aortic segments. The addition of hypercholesterolemic background to the elastase model, on the other hand, failed to exacerbate the lesion progression and rupture risk. Herein, we have summarized the distinct features of each model in Table 1. Further efforts are warranted to explore the optimal combinations to establish the desirable rAAA disease course in murine models.

**Table 1:** The distinct features of each model.

**Model**	**Procedure**	**End-point**	**Incidence rate of aneurysm**	**Special genetic background**	**Dietary modifications**	**Gross** **morphological** **features**	**Histological** **features**	**Ruptured AAA**
AngII-induced model (11)	AngII osmotic pump subcutaneous implantation	2–4 weeks	60~100% (63)	ApoE-/-;LDLr-/-	High fat/salt diet	AD, suprarenal AAA (11, 14)	Adventitial thickening and fibrosis, leukocyte infiltration, intima tear, ECM degradation, VSMC death, atherosclerotic lesions (with hypercholesterolemia)	15–30%, most in the acute phase (20, 21)
AngII+BAPN (Cooper et al. 2020; Kanematsu et al. 2020)	AngII osmotic pump subcutaneous implantation, together with BAPN administration (osmotic pump infusion or water supplementation)	4–6 weeks	30~49% (24)	None	Normal/high fat/high salt;	AD, AAA	Adventitial thickening and fibrosis, leukocyte infiltration, intima tear, intramural thrombosis, ECM degradation, VSMC death, atherosclerotic lesions (with hypercholesterolemia)	33–79% (24)
Intraluminal perfusion of PPE (42)	Pressure-perfusion of Type I porcine pancreatic elastase at 100mmHg	2–4 weeks	~100%	None	None	Infrarenal AAA	Adventitial thickening and fibrosis, media degeneration, intramural thrombosis, leukocyte infiltration, luminal expansion, ECM degradation, VSMC death	Rarely
Topical application of PPE (47)	Topical, peri-adventitial application of elastase to abdominal aortic segments	2 weeks	80%~87.5%	None	None	Small AAA	Adventitial thickening and fibrosis, leukocyte infiltration, luminal expansion, ECM degradation, VSMC death	Rarely
Combination of BAPN and topical elastase application (43)	BAPN-supplemented water modification and topical, peri-adventitial application of elastase	2–4 weeks	~95%	None	None	AD and AAA. Irregular shape, and/or iliac, renal arteries involvement	Intraluminal thrombosis, adventitial thickening and fibrosis, leukocyte infiltration, luminal expansion, ECM degradation, VSMC death	Two peaks of rupture:15.4% at first 2 weeks; 30.8% at chronic stage
Elastase perfusion+AngII (51)	Pressure-perfusion of elastase combined with AngII pump implantation	4 weeks	~100%	None	None		Adventitial thickening and fibrosis, leukocyte infiltration, luminal expansion, intima tear, intramural thrombosis, ECM degradation, VSMC death	60%
Calcium chloride/calcium phosphate induced model (52)	Adventitia application of calcium salts	2~4 weeks	~100%	None	None	Smaller AAA lesions. No thrombus. No progressive dilation of aneurysmal aortas.	Vascular calcification, adventitial thickening and fibrosis, inflammatory cell infiltration, oxidative stress, neovascularisation, elastin degradation, and VSMC death.	None

One key feature that is missing in all existing murine models is the gender dimorphism concerning aneurysmal rupture. For many models, e.g., AngII and elastase+BAPN models, female mice tend to develop aneurysms with smaller diameters than male, which aligns with epidemiological evidence in AAA patients. However, the rupture risk is also different between men and women. The dependence of growth and rupture rates on AAA diameter was similar in male and female patients, but the absolute rupture risk was 4-fold greater for all AAA sizes for female patients (64). Women with largest aneurysm diameter and smallest body size were at greatest rupture risks (65). Unfortunately, while most murine models could recapitulate the reduced AAA lesion size and incidence in female, the accelerated aneurysmal rupture risk has not yet been established in any of the existing models (36, 66).

Another major limitation concerning murine studies of rAAA lies in the lack of evaluation approaches. As of now, post-mortem examination remains the standard methodology to identify the presence and exact location of a bona fide rupture. Indeed, the onset of rAAA is highly unpredictable and often rapidly leads to mortality; therefore, live monitoring of rAAA is highly difficult to achieve from a utility perspective. Dissection microscopy is the most commonly used method for post-mortem examination of aortic ruptures, but the carcass quality as well as the experience level of the operators may influence the result. In recent studies, other post-mortem modalities such as nanoparticle contrast-enhanced computed tomography and high-resolution synchrotron-based x-ray have been utilized to determine aortic ruptures in murine models (59, 67). Future efforts are warranted to develop alternative strategies that could monitor or robustly predict the rupturing event in experimental models of AAA.

Feasibility is also a significant factor that should be considered in the choice of animal models. From the perspective of technical difficulties, the elastase perfusion model is undoubtedly the most challenging one. However, recent studies suggest that a topical, peri-aortic route of elastase application may provide similar outcomes in AAA disease progression and rupture, while considerably less technically demanding than the intraluminal route(49, 50). For AngII-based models, the prerequisite of a hypercholesterolemic background (e.g., ApoE-/- or LDLr-/-) dramatically increases the time and animal numbers needed, considering the breeding and expansion of double and sometimes even triple transgenic colonies. This is particularly a concern in terms of compliance with the 3Rs principle (replacement, reduction, refinement). The introduction of alternative methodologies such as viral PCSK9 gain-of-function and BAPN modification offers significant advantages in these regards, hence should be considered during the study designing phase.

In conclusion, thus far there are no single experimental models that faithfully recapitulate the disease features of rAAA. For future preclinical investigations, further modifications are still warranted to improve the clinical relevance of existing murine models.

## Author contributions

LY, EK, and BW conceived the manuscript, conducted the literature search, contributed to the drafting, and editing of the manuscript. All authors contributed to the article and approved the submitted version.

## Funding

This work was supported by the National Institute of Health (NIH) grant R01HL162895 (to BW).

## Conflict of interest

The authors declare that the research was conducted in the absence of any commercial or financial relationships that could be construed as a potential conflict of interest.

## Publisher's note

All claims expressed in this article are solely those of the authors and do not necessarily represent those of their affiliated organizations, or those of the publisher, the editors and the reviewers. Any product that may be evaluated in this article, or claim that may be made by its manufacturer, is not guaranteed or endorsed by the publisher.
